# The impact of mind–body therapies on the mental health of women victims of violence: A meta-analysis

**DOI:** 10.1007/s00737-024-01484-8

**Published:** 2024-07-03

**Authors:** Sevgi Koroglu, Gülgün Durat

**Affiliations:** Faculty of Health Sciences, Department of Psychiatric Nursing, Sakarya University, Sakarya, Turkey

**Keywords:** Mind–body therapies, Complementary therapies, Gender-based violence, Intimate partner violence, Domestic violence

## Abstract

**Purpose:**

Violence against women is a common public health problem and causes negative mental health outcomes. Mind–body therapies aim to positively affect a person's mental health by focusing on the interaction between mind, body, and behavior. Therefore, this study aims to evaluate the effect of mind–body therapies on women's mental health.

**Methods:**

Randomized controlled trials published in the last 20 years comparing mind–body therapies with active control or waiting lists in women victims of violence were included. Pubmed, Cochrane, Scopus, Web of Science, and CINAHL databases were searched until August 2023. The random effects model and fixed effects model were used for data analysis. The heterogeneity of the study was assessed using the I^2^ index, and publication bias was assessed using Egger's test and funnel plot.

**Results:**

Twelve eligible studies with a sample size of 440 women victims of violence were selected. Mind–body therapies led to a statistically significant reduction in anxiety scores (SMD: 1.95, 95% CI: 1.01, 2.89), depression scores (SMD: 1.68, 95% CI: 0.83, 2.52) and posttraumatic stress scores (SMD: 0.95, 95% CI: 0.73, 1.18). There was a high level of heterogeneity in the outcome for anxiety (I^2^ = 85.18), a high level of heterogeneity for depression (I^2^ = 88.82), and a low level of heterogeneity for PTSD (I^2^ = 19.61). Results of subgroup analysis based on the number of sessions showed that eight or fewer sessions reduced anxiety (SMD: 3.10, 95% CI: 1.37, 4.83) and depression scores (SMD: 3.44, 95% CI: 1.21, 5.68), while PTSD scores did not change.

**Conclusion:**

Evidence suggests that mind–body therapies may reduce anxiety, depression, and PTSD in women victims of violence.

## Introduction

Violence against women is recognized as an important public health problem and a violation of human rights (Oram et al. 2017). According to the World Health Organization's report covering the years 2000 and 2018, approximately one-third or 30% of women worldwide have been subjected to physical and/or sexual violence by a partner, a non-partner, or both (World Health Organization 2021). In Turkey, according to 2014 data, 8.2 percent of women have been subjected to physical violence, 5.3 percent to sexual violence, and 25.7 percent to emotional violence (Turkish Statistical Institute 2021). According to a study conducted in Brazil in 2023, the lifetime prevalence of physical violence against women was found to be 22.4% (Nakamura et al. 2023). According to 2021 data in Ethiopia, the prevalence of intimate partner violence in pregnant women is 39.2%. 29.8% of pregnant women suffer from physical, 26.8% from sexual, and 22.2% from emotional intimate partner violence (Abebe et al. 2022). In a study conducted in China in 2017, intimate partner violence during pregnancy was found to be 7.7% (Wang et al. 2017).

The concept of "women victims of violence" refers to women who have been subjected to physical, sexual, and psychological forms of violence within the family, by intimate partners, or by the community in various contexts. This victimization is based on factors such as power imbalances in gender relations, low socio-economic status, oppressive social norms, and the lack of legal and policy arrangements to protect women (Campbell and Mannell 2016; Yari et al. 2021). These factors fuelling gender-based violence are also compounded by a lack of political will to implement pro-women policies in male-dominated criminal and civil justice systems (Campbell and Mannell 2016). Women victims of violence avoid telling others about their experiences of abuse due to emotional reactions such as shame, fear, guilt, desire to protect the perpetrator, stigmatisation, social and cultural norms (Boethius and Åkerström 2020). This reluctance may cause the problem to deepen by preventing women from seeking help and reporting the perpetrator (Mulaudzi et al. 2022). Continued exposure to inhumane and degrading acts against women reveals an increased risk of mental disorders (Oram et al. 2017). Women victims of violence have depressive symptoms, anxiety, post-traumatic stress disorder (PTSD), irritability, behavioural problems, memory and concentration difficulties, sleep and eating disorders, suicidal ideation, low self-esteem, alcohol and substance abuse (World Health Organization 2005). These negative effects are not limited to periods of increased frequency and intensity of violence. Victims of violence continue to be exposed to negative psychological effects for years due to traumatic experiences (Navarro-Mantas et al 2021). Due to the storage of the traumatic event as procedural memory, the trauma can be felt as if it is being experienced again. When traumatic memories are activated by the emotion, sound, or image reminiscent of the memory, a somatic experience (such as tachycardia, sweating, trembling, headache, nausea, or muscle tension) may be experienced (Langmuir et al. 2012). Somatic reactions that occur when the traumatic memory is activated can increase the severity of PTSD symptoms (McFarlane et al. 1994). When PTSD symptoms exacerbated by somatic reactions are considered, the importance of practices that increase the individual's awareness of the changes occurring in the body and help to manage them increases (Nguyen-Feng et al. 2019). Post-traumatic symptoms, depression, and anxiety can be relieved with various mental health treatments applied to women victims of violence. Cognitive behavioral therapies (CBT) can be used for female victims (Arroyo et al. 2017; Crespo et al. 2022; Nemeroff et al. 2006). Cognitive or cognitive-behavioral interventions for PTSD; cognitive, cognitive-behavioral, and behavioral interventions for depression; and cognitive-behavioral interventions for anxiety are effective (Trabold et al. 2018). Although the positive effect of CBT on PTSD symptoms is recognized, dropout problems may occur in therapy (Gutner et al. 2016; Najavits 2015). Moreover, these treatments aim to improve mental health, without directly targeting physical health (Pebole et al. 2021). Thus, low-cost, low-risk alternative interventions that offer a holistic approach for individuals who cannot adapt to traditional therapies in the treatment of PTSD-related symptoms are gaining importance (Zhu et al. 2022).

Mind–body therapies (MBT) emphasize the importance of the mind for well-being. Focusing on the interaction between mind, body, and behavior, MBTs are used to promote health through the mind and improve physical health (Ramirez-Garcia et al. 2019). MBTs differ from other types of therapy in that they focus on the interconnectedness of mind and body and emphasize the influence of emotional, behavioral, spiritual, and social factors on physical health and symptoms (Landier and Tse 2010). MBTs include Tai Chi, Qigong, body-focused psychotherapy, yoga, mindfulness-based therapies, meditation, and relaxation exercises (Fogaça et al. 2021; Ramirez-Garcia et al. 2019). MBTs are effective in reducing symptoms of anxiety or depression in individuals with chronic illness or mental disorders, pregnant or infertile women, and populations such as veterans and refugees with traumatic experiences (Birling et al. 2021; Gaitzsch et al. 2020; Lynch et al. 2018; Marc et al. 2011; Vancampfort et al. 2021). Hyperactivation of the autonomic nervous system caused by emotional stress is known to elicit physical symptoms and negatively affect the prognosis of medical conditions (Jacobs 2001). MBTs have been suggested to produce therapeutic effects by reducing sympathetic nervous system and cerebral cortical activation (Taylor et al. 2010). Impaired neuroplasticity resulting from adverse life events is held responsible for the development of anxiety, depression, and PTSD (Arnetz et al. 2020). Impaired neuroplasticity can be improved by using appropriate treatment and rehabilitation methods (Holtzheimer et al. 2019). Evidence that meditation thickens brain regions related to attention, proprioception, and sensory processing, and mindfulness-based stress reduction increases grey matter concentration in brain regions related to learning and memory processes, emotion regulation, self-referential processing, and perspective taking have shown the beneficial neuroplasticity potential of MBTs, suggesting that MBTs may provide long-term efficacy (Hölzel et al. 2011; Lazar et al. 2005). MBTs positively affect quality of life, including energy levels. It is observed that individuals who apply these therapies experience an increase in vitality, alertness, and activity levels in their daily lives (Oken et al. 2006; Sirois et al. 2018). The positive effect of MBTs also includes physical health. MBTs are associated with an increase in muscle flexibility, endurance, and strength, and improvement in cardiopulmonary endurance. They also have positive effects such as helping to relieve pain, strengthening digestion and immunity, and improving sleep quality (Shin 2021; Zheng et al. 2017). Beyond physical well-being, MBTs have positive effects on mental health. While effectively reducing anxiety, depression, anger, and burnout levels, they lead to higher social functioning (Chobe et al. 2020; Lynch et al. 2018; Oken et al. 2006; Sirois et al. 2018). Systematic reviews and meta-analyses support the effectiveness of MBTs in addressing mental health problems. Studies involving various populations have shown improvements in PTSD symptoms, depression, and anxiety following participation in MBTs (Cramer et al. 2017, 2018; Kim et al. 2013; Li et al. 2019). Yoga has provided significant reductions in stress, physical complaints (Beranbaum and D'Andrea 2023), PTSD, and dissociative symptomatology in traumatized women (Price et al. 2017). Although there is limited evidence in the literature that MBTs improve the mental health outcomes of women victims of violence, there is significant evidence of their effectiveness in different populations (Cramer et al. 2017, 2018; Kim et al. 2013; Li et al. 2019). There is a need to understand the effectiveness of MBTs that focus on the physical symptoms of trauma, which can provide long-term effectiveness with beneficial neuroplasticity, in women victims of violence.

This study aims to provide a synthesis of evidence from randomized controlled trials published in the last two decades to determine the effectiveness of different mind–body therapies (e.g. yoga, meditation, mindfulness-based therapies) on the mental health of women victims of violence.

## Method

This research is a systematic review and meta-analysis. The preparation of the study protocol and reporting of the manuscript was carried out according to the Preferred Reporting Items for Systematic Reviews and Meta-Analyses (PRISMA) (Page et al. 2021). Before the review, the study protocol was registered in the International Prospective Register of Systematic Reviews (PROSPERO) with accession number CRD42023448993.

## Research question

In the current systematic review and meta-analysis, the PICOS tool was applied to develop a search strategy by PRISMA recommendations: (P) Population: Adult women aged 18 years and older with a history of exposure to any form of violence; (I) Intervention: relaxation exercises, progressive muscle relaxation exercise, yoga, pranayama breathing, breathing exercises, pilates, Qigong, Tai Chi, imagery, meditation, mindfulness-based exercises, aromatherapy, hypnosis, laughter therapy, psychodrama, dance, biofeedback, body-focused therapies; (C) Comparison: active control or waiting list; (O) Outcome: depression, anxiety, and post-traumatic stress disorder (S) Study design: randomized controlled trials.

## Search strategy

Literature was searched in PubMed (MEDLINE), Cochrane, Scopus, Web of Science, and CINAHL databases for the last 20 years (2003–2023) between 15 July and 15 August 2023. ("violence against women" OR "gender-based violence" OR "gender violence" OR "gender-associated violence" OR "gender-related violence" OR "intimate partner violence" OR "domestic violence" OR "spouse abuse" OR "battered women" OR "family violence" OR "partner abuse" OR "maltreatment" OR "marital rape" OR "forced sex" OR "non- partner sexual violence" OR "physical abuse" OR "sexual abuse") AND ("relaxation" OR "yoga" OR "breathing" OR "pranayama" OR "mind body" OR "mind–body" OR "pilates" OR "qigong" OR "tai chi" OR "tai ji" OR "imagery" OR "meditation" OR "mindfulness" OR "aromatherapy" OR "hypnosis" OR "laughter therapy" OR "psychodrama" OR "progressive muscle" OR "dance" OR "biofeedback" OR "complementary therapies" OR "alternative therapies" OR "health promotion" OR "physical activity") keyword combination was used. Only articles in English were included.

## Eligibility criteria

The inclusion criteria for the analyses were as follows: 1) articles in English, 2) randomized controlled trials, 3) studies reporting data from women with a history of exposure to any form of violence, 4) studies with samples aged 18 years and over, 5) relaxation exercises, progressive muscle relaxation exercise, yoga, pranayama breathing, breathing exercises, pilates, Qigong, Tai Chi, imagery, meditation, mindfulness-based exercises, aromatherapy, hypnosis, studies describing mind–body therapies such as laughter therapy, psychodrama, dance, biofeedback, body-focused therapies as interventions, 6) studies reporting outcomes of depression, anxiety and post-traumatic stress disorder assessed by a valid and reliable scale, 7) studies reporting relevant outcomes as mean, standard deviation, 8) studies published in 2003 or later. Control groups could include any form of intervention, including a control intervention or usual care. The exclusion criteria for this analysis were 1) qualitative studies, correlation studies, cross-sectional studies, case–control studies, and cohort studies 2) the study population consisted of children or adolescents.

## Study selection

**Fig. 1:**
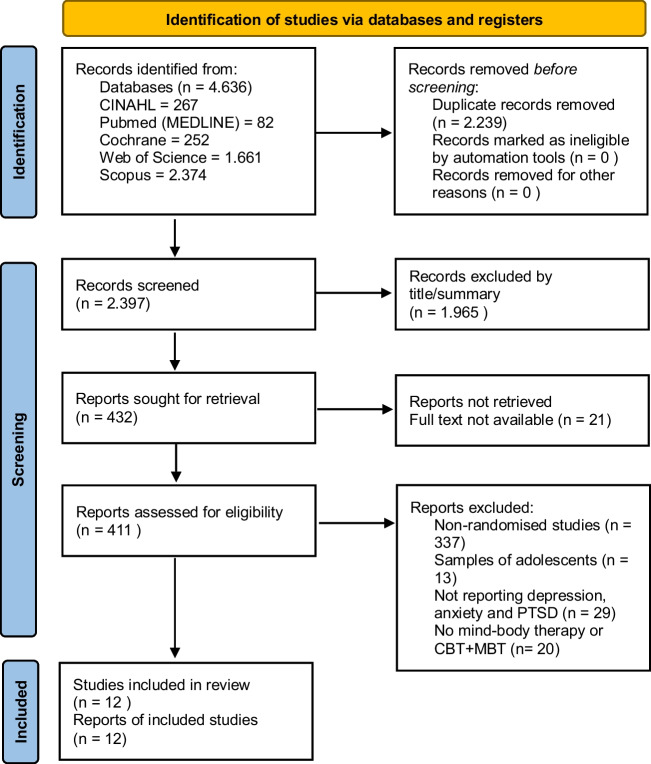
Diagram of selection of studies

All references obtained through the search strategy were exported with the reference management software Endnote X9 and duplicate references were removed. The identification of the studies to be included in this review was carried out independently by the researchers according to the inclusion and exclusion criteria. Firstly, title/abstract screening was carried out by two independent authors. Studies with titles/summaries that did not meet the inclusion criteria were excluded at this step. Eligible studies were saved in a template created in Excel so that the full text could be read and evaluated. This template consisted of study details (author, publication year, country), inclusion or exclusion status and reasons for exclusion. Full-text reviews of data extraction, methodological quality assessment and analysis of included studies were performed by two independent authors. Any disagreements were resolved by discussing the full text of the article. The PRISMA Flow Diagram of the study selection process is given in Fig. 1.

## Data extraction

Data extraction was carried out by two independent authors to obtain the research data. The information extracted included first author, year of publication, country, sample size and age, intervention and control group, type of intervention, characteristics of the intervention, measurement tools used and results.

To minimise the risk of bias, the literature search, selection of articles, data extraction and quality assessment of the articles were performed separately by two researchers. Any disagreements were resolved by discussing the full text of the article.

## Reporting quality

Joanna Briggs Institute critical appraisal tools were used for quality assessment of studies. "JBI Critical Appraisal Checklist for Randomised Controlled Trials" consisting of 13 items was used for quality assessment of randomised controlled trials (Barker et al. 2023). Each item in the checklists was coded as yes, no, uncertain or not applicable. In the checklist, items with "no" and "uncertain" responses received 0 points, while items with "yes" responses received 1 point. For the response "not applicable", it was coded as the criterion was not suitable for the study and could not be scored. Quality scores were given by two independent authors (GD and SK). There was 92.9% inter-rater agreement on the reporting of quality scores. All discrepancies were discussed and reconciled by the coders.

## *Meta*-analytic method

Stata 16.0 programme was used to perform the meta-analysis. Standardised mean differences (SMD), alpha value of 0.05 and 95% confidence interval (95% CI) were used for continuous variables. Heterogeneity between included studies was assessed by I^2^. An I^2^ statistic of 0.50 and above was considered as high heterogeneity and random effects model was used. If the I^2^ statistic is below 0.50, it is considered as low heterogeneity and fixed effects model is used. In this study, DerSimonian Laird method, random effects model and fixed effects model were used. Subgroup analysis was performed to determine the possible cause of heterogeneity. The size of the effects were defined as small (r = 0.1), medium (r = 0.3) and large (r = 0.5) effect size (Cohen 1988). The funnel plot and Egger test were used to evaluate the publication bias of the studies included in the meta-analysis (Dinçer 2014). When publication bias was detected, "leave one out" sensitivity analysis was applied. Tables were used to summarise the main characteristics of the included studies and a forest plot showing the effect size and confidence interval was used to present the results of the studies.

## Results

### Included studies

As a result of the search in electronic databases, 4,636 articles were found. After the duplicates were removed, 2,397 articles remained. After title/abstract screening, 411 articles were identified as review articles. Of these studies, 397 were excluded from the meta-analysis because they were not randomized controlled trials (n = 337), they worked with adolescent samples (n = 13), they did not report depression, anxiety, and PTSD outcomes (n = 29), the intervention was not MBT or was combined with CBT (n = 20) (Fig. 1). Twelve studies were included in the meta-analysis.

### Characterization of studies

The studies included in the meta-analysis include five countries. These studies were conducted in the United States (Franzblau et al. 2008; Gallegos et al. 2020; Hernández-Ruiz 2005; Kelly and Garland 2016; Lee et al. 2017; Özümerzifon et al. 2022; van der Kolk et al. 2014), Australia (Leach and Lorenzon 2023), Sweden (Rudstam et al. 2022), Canada (Classen et al. 2020) and Iran (Ghahari et al. 2017; Goodarzi et al. 2020). Among the included studies, 66.7% (n = 8) provided information on race or ethnicity, 33.3% (n = 4) on income, 83.3% (n = 10) on education, 58.3% (n = 7) on employment status, and 66.7% (n = 8) on marital status. A total of 66.7% (n = 8) of the studies specified the context of violence. 4 of the studies defined IPV (Franzblau et al. 2008; Gallegos et al. 2020; Kelly and Garland 2016; Özümerzifon et al. 2022), 3 defined domestic violence (Ghahari et al. 2017; Hernández-Ruiz 2005; Leach and Lorenzon 2023), and 1 defined interpersonal violence (Lee et al. 2017). Among the studies, 58.3% (n = 7) reported the type of violence. Among the studies, 1 identified sexual violence (Goodarzi et al. 2020), 3 identified physical or sexual violence (Gallegos et al. 2020; Kelly and Garland 2016; Rudstam et al. 2022), 1 identified verbal or physical violence (Hernández-Ruiz 2005), 1 identified physical, sexual or emotional violence (Lee et al. 2017) and 1 identified verbal, emotional, physical or sexual violence (Franzblau et al. 2008). There were 440 participants in total, 219 participants in the mind–body therapy group and 221 participants in the control group (active control or waiting list). The largest sample was 64 participants (van der Kolk et al. 2014) and the smallest sample was 16 participants (Goodarzi et al. 2020). The duration of mind–body interventions ranged from 5 days to 12 weeks. The number of sessions of the interventions ranged from 2 to 12 sessions, with session durations ranging from 30 to 150 min each time. Of the 12 eligible studies, 3 were dance or music therapy (Hernández-Ruiz 2005; Özümerzifon et al. 2022; Rudstam et al. 2022), 2 were meditation (Leach and Lorenzon 2023; Lee et al. 2017), 2 were yoga or yogic breathing (Franzblau et al. 2008; van der Kolk et al. 2014), 4 included mindfulness (Gallegos et al. 2020; Ghahari et al. 2017; Goodarzi et al. 2020; Kelly and Garland 2016) and 1 included body-focused sensory motor psychotherapy (Classen et al. 2020). When the measurement tools evaluating anxiety symptoms were examined, 2 of the studies used State-Trait Anxiety Inventory (Ghahari et al. 2017; Hernández-Ruiz 2005), 2 of the studies used Beck Anxiety Inventory (Classen et al. 2020; Goodarzi et al. 2020), 1 of the studies used Hopkins Symptom Checklist-25 (Rudstam et al. 2022) and 1 of the studies used Depression Anxiety Stress Scale (Leach and Lorenzon 2023). In order to measure depression symptoms, 6 of them used Beck Depression Inventory-II (Classen et al. 2020; Franzblau et al. 2008; Ghahari et al. 2017; Goodarzi et al. 2020; Kelly and Garland 2016; van der Kolk et al. 2014), 1 of them used Hopkins Symptom Checklist-25 (Rudstam et al. 2022), and 1 of them used Depression Anxiety Stress Scale (Leach and Lorenzon 2023) (Table 1).

**Table 1:** Summary characteristics of included studies

Authors, publishing year, country	Participants	Age (Mean ± SD)	Intervention Program	Training	Outcome Measured	Follow-up	Results
Özümerzifon et al. 2022, USA	43 women survivors of intimate partner violence	I = 35 ± 6 C = 38 ± 8	I = Dance/movement workshop program + usual care (n: 25) C = Usual care (n: 18)	12 sessions of 90 min per session Duration (week): 6	PCL-5, K6 + Distress Scale	6 weeks	No significant change was observed in the K-6 + scale results The PCL-5 total score showed a significant time effect (F(1) = 15.52, p < 0.001, η^2^ = 0.275), decreasing significantly over the intervention period
Leach and Lorenzon 2023, Australia	42 women victims of domestic violence	I = 48.2 ± 12.5 C = 47.4 ± 12.4	I = Transcendental meditation (n: 21) C = Group support (n: 21)	9 sessions of 1–2 h per session Duration (week): 8	PCL-5, DASS-21, AQoL8D	1) 8 weeks 2) 16 weeks	Transcendental meditation was found to be significantly more effective than group support in improving depression (p = 0.029) and anxiety (p = 0.017) severity scores
Rudstam et al. 2022, Sweden	40 women with a history of sexual or physical abuse	I = 45.2 ± 10.7 C = 42.2 ± 9.10	I = Music therapy + imagery (n: 21) C = Waitlist (n:19)	12 sessions of 150 min per session Duration (week): 12	PCL-5, PSOMS, DES, SDQ-5, HSCL-25	1) 12 weeks 2) 3 months	The interaction between state and time for the PCL-5 total score showed a significant change in the intervention group compared to the control group (F(1, 42) = 8.68, p = .005, d = 0.94). It showed large interaction with d = 0.87 for anxiety and moderate effects with d = 0.67 for depression
Van der Kolk et al. 2014**,** USA	64 women with chronic, treatment-resistant post-traumatic stress disorder	I = 41.5 ± 12.2 C = 44.3 ± 11.9	I = Yoga (n: 32) C = Women’s health education (n: 32)	10 sessions of 60 min per session Duration (week): 10	CAPS, DES, BDI-II, Davidson Trauma Scale, Inventory of Altered Self Capacities	1) 5 weeks 2) 10 weeks	Yoga treatment showed positive effects on CAPS, Davidson Trauma Scale, BDI-II. The yoga group showed more significant improvement, especially in the reduction of PTSD symptoms and stress reduction
Hernández-Ruiz 2005**,** USA	28 women subjected to physical or verbal violence in a domestic violence shelter	Mean age of the sample = 35.36	I = Music therapy (n: 14) C = Silence (n: 14)	5 sessions of 30 min per session Duration (day): 5	PSQI, STAI	4 days	Music therapy was significant both in the main effect modification of anxiety level (F(1, 26) = 17.68, p < .001) and in an interaction by condition (F(1, 26) = 15.73, p = .001)
Kelly and Garland 2016, USA	39 women victims of physical or sexual violence with a history of intimate partner violence	Mean age of the sample = 41.5 ± 14.6	I = Trauma-informed model of mindfulness-based stress reduction (n: 20) C = Waitlist (n: 19)	8 sessions of 2–2,5 h per session Duration (week): 8	RSQ, PCL-C, BDI-II	8 weeks	Relative to the control group, participation in the TI-MBSR was associated with statistically and clinically significant reductions in PTSD and depressive symptoms
Lee et al. 2017, USA	58 women victims of physical, sexual or emotional violence who have experienced interpersonal violence	I = 39 ± 9 C = 38.1 ± 8.2	I = Meditation + usual care (n: 32) C = Usual care (n: 26)	5 days a week, 1 h twice a day for a total of 60 h Duration (week): 6	SDS, MPSS	6 weeks	The intervention group showed significant changes in trauma symptoms (t = 6.009, df = 31, p = .000) from pre-treatment to post-treatment, while no significant changes were observed in the control group
Classen et al., 2020, Canada	23 women with a history of childhood abuse	Mean age of the sample = 43.51 ± 10.01	I = A sensory motor psychotherapy called the Trauma and Body Group (n: 13) C = Waitlist (n: 10)	20 sessions Duration (week): Not reported	SBC, PCL-C, SDQ-20, DES, BAI, BDI-II, PHLMS, SRS	1) First follow-up (Time not reported) 2) Second follow-up (Time not reported)	It was found that the intervention provided significant improvements in anxiety (p = 0.02) score
Ghahari et al. 2017, Iran	30 women victims of domestic violence	Mean age of the sample = 36.6	I = Mindfulness-based cognitive therapy (n: 15) C = Waitlist (n: 15)	8 sessions of 45 min per session Duration (week): Not reported	BDI-II, STAI	Post-test (Time not reported)	The intervention was found to reduce depression and anxiety of women victims of violence (p < 001)
Goodarzi et al. 2020, Iran	16 women victims of sexual assault	I = 32.6 ± 9.1 C = 29.1 ± 6.3	I = Art therapy + mindfulness (n: 8) C = No intervention (n: 8)	8 sessions of 2 h per session Duration (week): 8	BDI-II, BAI, PFQ-2	1) 8 weeks 2) Follw-up (Time not reported)	The intervention was effective in reducing symptoms of depression and anxiety at post-test and follow-up compared to untreated controls
Gallegos et al. 2020, USA	Victims of intimate partner violence, physical or sexual violence17 women	Mean age of the sample = 42.69 ± 13.11	I = Mindfulness-based stress reduction (n: 8) C = Health education guidelines (n: 9)	8 sessions of 2 h per session Duration (week): 8	PCL-5, DERS, UFOV	1) 8 weeks 2) 12 weeks	While a statistically significant decrease in PTSD symptoms was observed after the intervention, F(1.37, 16.53) = 5.19, p < .05, this decrease was not observed after the control intervention
Franzblau et al. 2008, USA	40 women victims of intimate partner violence, including verbal, emotional, physical or sexual violence	18 to 45 years	C = Testimony (n: 10) I = Yogic breath (n: 10) C = Testimony + yogic breath (n: 10) C = No intervention (n: 10)	Testimony: 2 sessions of 45-min per session Yogic breath: 2 sessions of 45-min per session Testimony + yogic breath: 4 sessions of 45-min per session	BDI-II	4 days	Learning yoga breathing techniques alone and in combination with witnessing has shown to significantly reduce feelings of depression

PTSD: Post-Traumatic Stress Disorder, I: Intervention group, C: Control group, PCL-5: The Posttraumatic Stress Disorder Checklist-DSM IV, DASS-21: Depression Anxiety Stress Scale, AQoL8D: Australian Quality of Life – 8 dimension, PSOMS: Positive States of Mind Scale, DES: Dissociative Experience Scale, SDQ-5: Somatic Dissociation Questionnaire – 5 items, HSCL-25: The Hopkins Symptom Checklist-25, CAPS: The Clinician Administered Post-Traumatic Stress Disorder Scale, BDI-II: Beck’s Depression Inventory-II, PSQI: The Pittsburgh Sleep Quality Index, STAI: State Trait Anxiety Inventory, RSQ: The Relationship Structures Questionnaire, PCL-C: Post-Traumatic Stress Disorder Checklist-Civilian Version, SDS: Symptom Distress Scale, MPSS: Modified Post-Traumatic Stress Disorder Symptom Scale, SBC: Scale of Body Connection, SDQ-20: Somatoform Dissociation Questionnaire, BAI: Beck Anxiety Inventory, PHLMS: Philadelphia Mindfulness Scale, SRS: Soothing Receptivity Scale, PFQ-2: The Personal Feelings Questionnaire-2, DERS: Difficulties in Emotion Regulation Scale, UFOV: Useful Field of View Test

## Effect sizes

### Anxiety symptoms

**Fig. 2:**
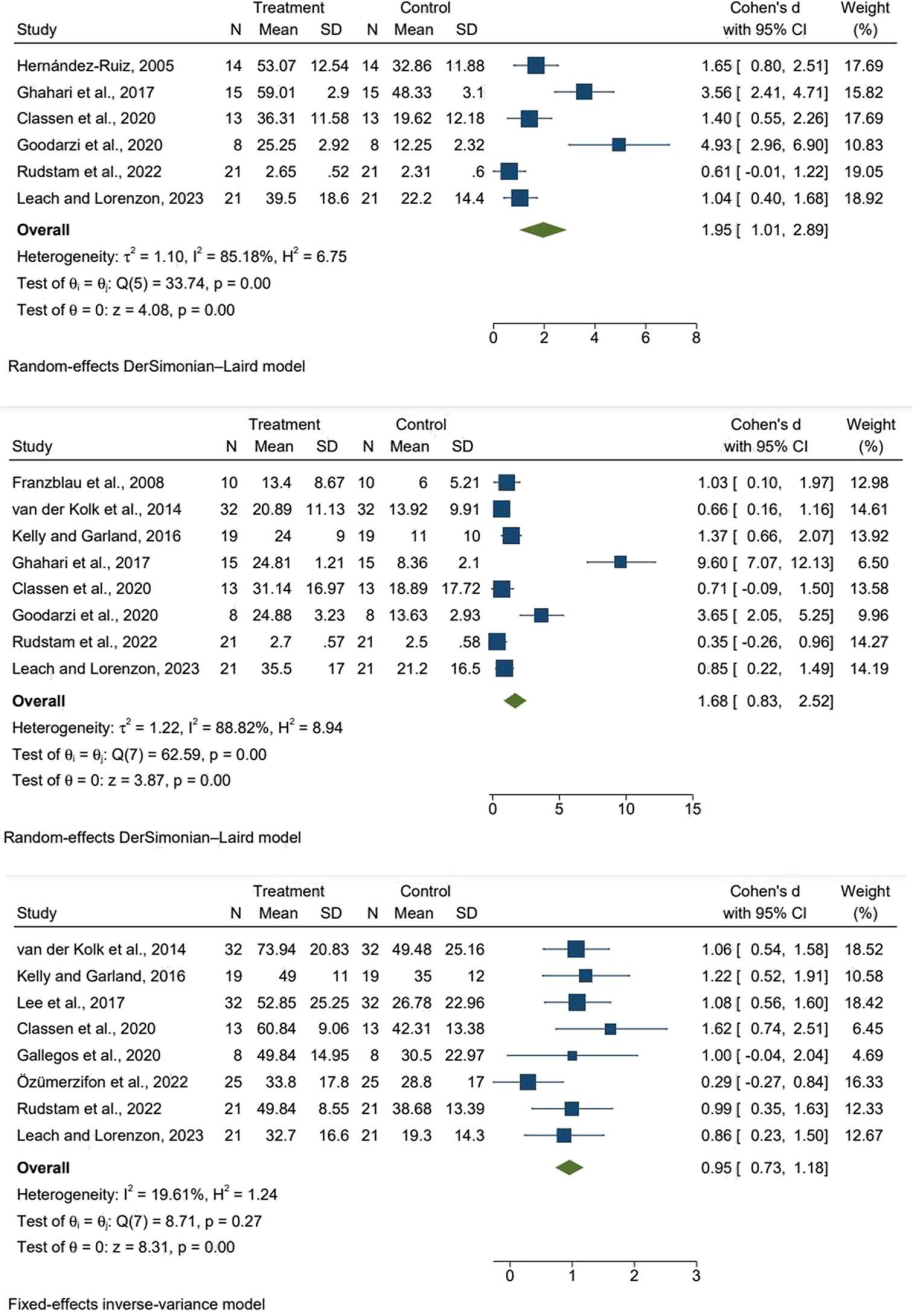
Forest plots of the relationship between mind–body therapy and mental health outcomes

We included 6 studies (n = 179) reporting anxiety as a mental health outcome. The analysis revealed a significant effect and high heterogeneity (effect size [SMD = 1.95 (95% CI 1.01 to 2.89), p = 0.00; I^2^ = 85.18%]). MBTs had an large effect on reducing anxiety scores than active control or wait-list (Fig. 2).

### Depressive symptoms

We included 8 studies (n = 294) reporting depression as a mental health outcome. The analysis revealed a significant effect and high heterogeneity (effect size [SMD = 1.68 (95% CI 0.83 to 2.52), p = 0.00; I^2^ = 88.82%]). MBTs had an large effect in reducing depression scores than active control or wait-list (Fig. 2).

### PTSD symptoms

We included 8 studies (n = 326) that reported PTSD as a mental health outcome. Analysis revealed a significant effect and low heterogeneity (effect size [SMD = 0.95 (95% CI 0.73 to 1.18), p = 0.00; I^2^ = 19.61%]). MBTs had a large effect on the reduction of PTSD scores than active control or wait-list (Fig. 2).

### Subgroup analyze

**Fig. 3:**
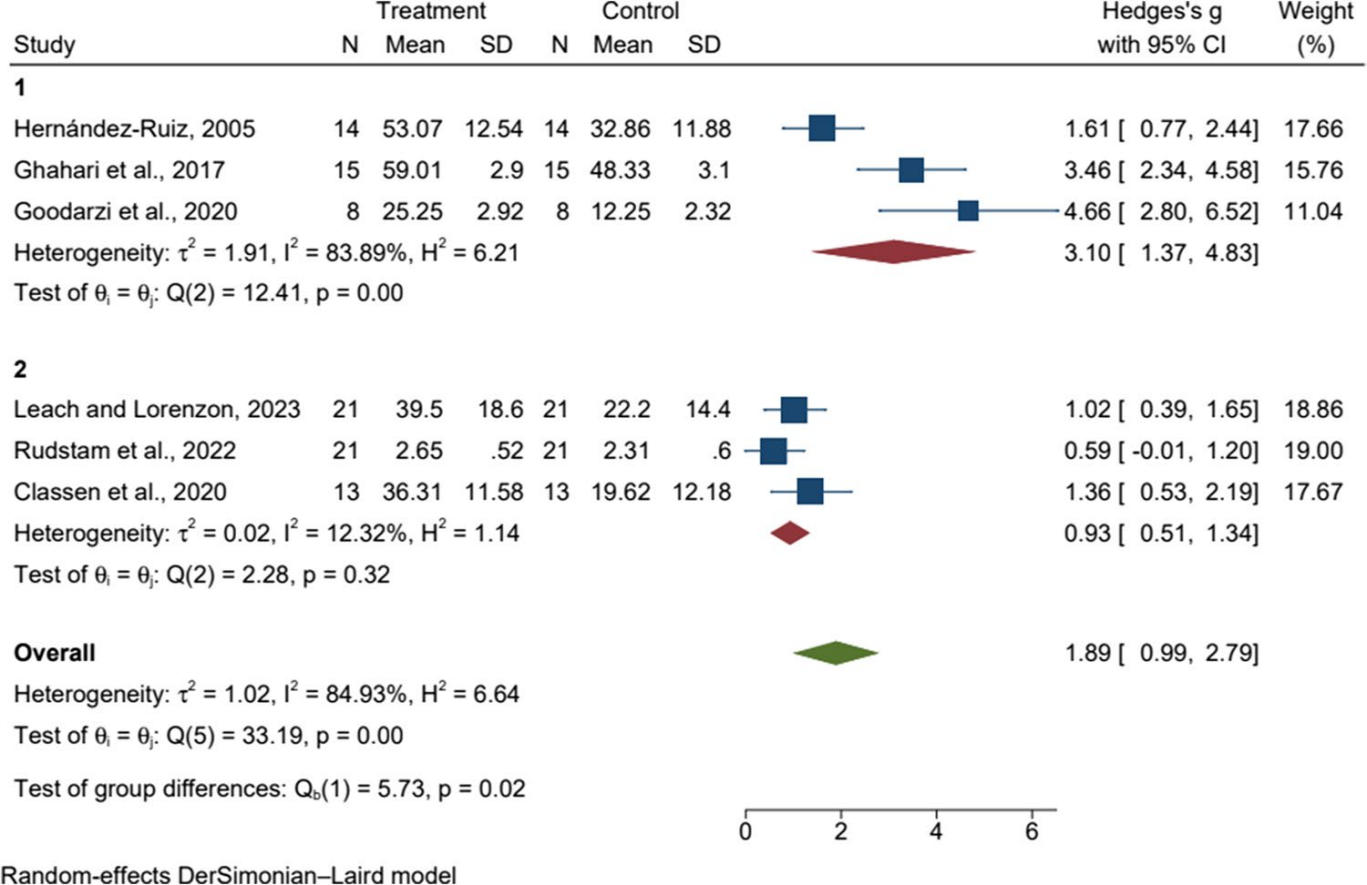
Forest plot of the relationship between the number of mind–body therapy sessions and anxiety 1: The number of sessions is eight or less, 2: The number of sessions is more than eight

**Fig. 4:**
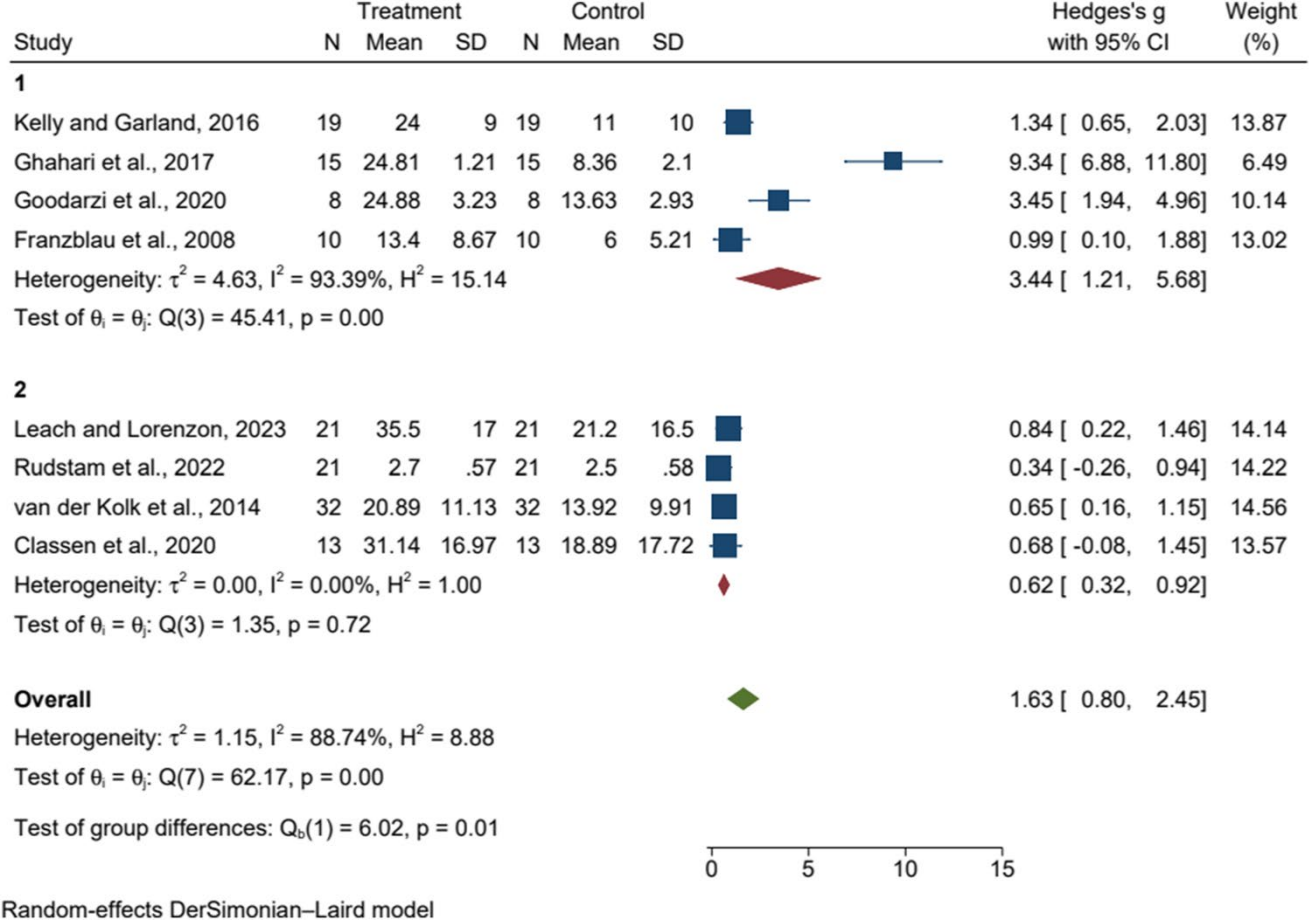
Forest plot of the relationship between the number of mind–body therapy sessions and depression1: The number of sessions is eight or less, 2: The number of sessions is more than eight

**Fig. 5:**
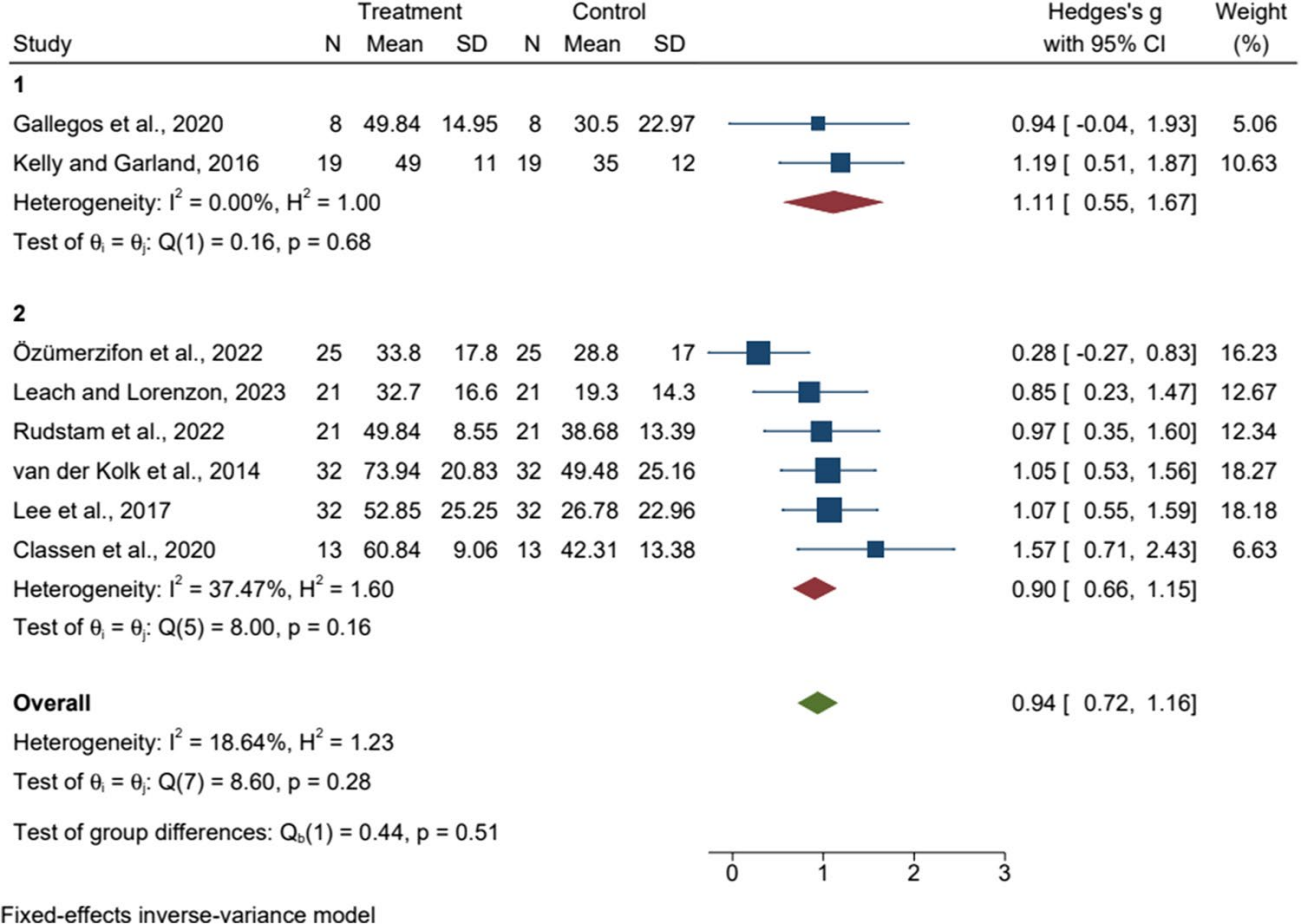
Forest plot of the relationship between the number of mind-body therapy sessions and PTSD

All eligible articles were categorized according to the number of sessions as shown in Figs. 3, 4, and 5. In subgroup analyses by number of sessions for anxiety, there was significant heterogeneity between studies (p = 0.00, I^2^ > 50%), so we used a random effects model for analysis. The results showed that eight or fewer MBT sessions were effective and statistically significant in reducing anxiety scores compared to active control or wait-list [SMD = 3.10, 95%CI (1.37, 4.83), p = 0.00]. In subgroup analyses by number of sessions for depression, there was significant heterogeneity between studies (p = 0.00, I^2^ > 50%), so we used the random effects model for analysis. The results showed that eight or fewer MBT sessions were effective and statistically significant in reducing depression scores compared to active control or wait-list [SMD = 3.44, 95%CI (1.21, 5.68), p = 0.00]. In the subgroup analysis by number of sessions for PTSD, there was low heterogeneity between studies (p = 0.28, I^2^ = 18.64%), so we used a fixed effects model for the analysis. PTSD scores did not show statistically significant improvements by number of sessions.

## Publication bias

**Fig. 6:**
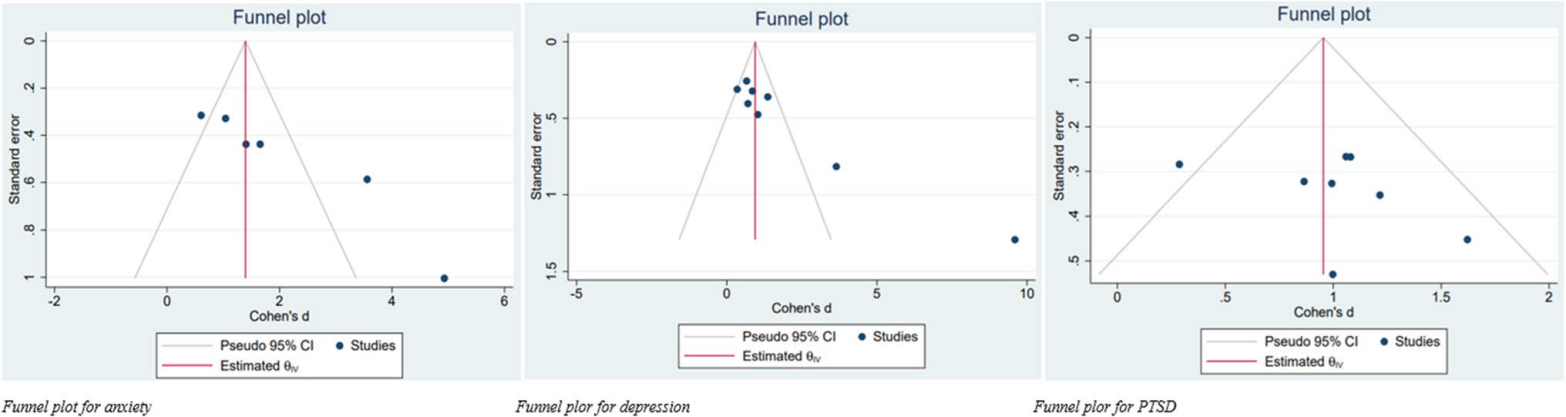
Funnel plot

The funnel plot and Egger's test were used to assess publication bias (Fig. 6). Although funnel plots for MBTs and PTSD symptoms showed symmetry, funnel plots for MBTs and anxiety and depressive symptoms showed asymmetry. As a result of Egger's test, p = 0.00 for anxiety and depression and p = 0.29 for PTSD. Publication bias was detected for anxiety and depression. "Leave one out" sensitivity analysis was performed for all groups with an I^2^ > 50%, and the pooled results did not change substantially.

## Reporting quality

The methodological quality scores of all studies included in the systematic review ranged from 5 to 11 (Table 2). The methodological quality of the two studies was below 50% (Classen et al. 2020; Ghahari et al. 2017). Only 3 studies had blinded outcome assessors (Kelly and Garland 2016; Leach and Lorenzon 2023; van der Kolk et al. 2014).

**Table 2:** Quality assessment of the included studies

Authors, publishing year, country	Item 1	Item 2	Item 3	Item 4	Item 5	Item 6	Item 7	Item 8	Item 9	Item 10	Item 11	Item 12	Item 13	Score
Özümerzifon et al. 2022,USA	1	1	1	0	0	0	1	1	0	1	1	1	1	9
Leach and Lorenzon 2023,Australia	1	1	1	0	0	1	1	1	1	1	1	1	1	11
Rudstam et al. 2022, Sweden	1	1	1	0	0	0	0	1	0	1	1	1	1	8
Van der Kolk et al. 2014, USA	0	0	1	0	0	1	1	1	1	1	1	1	1	9
Hernández-Ruiz 2005, USA	0	1	0	0	0	0	1	1	1	1	0	1	1	7
Kelly and Garland 2016, USA	0	0	1	0	0	1	0	1	1	1	1	1	1	8
Lee et al. 2017, USA	1	1	1	0	0	0	1	1	0	1	1	1	1	9
Classen et al., 2020, Canada	0	0	1	0	0	0	0	0	1	1	1	1	1	6
Ghahari et al. 2017, Iran	0	0	0	0	0	0	0	1	1	1	0	1	1	5
Goodarzi et al. 2020, Iran	0	0	1	0	0	0	1	1	1	1	1	1	1	8
Gallegos et al. 2020, USA	1	1	0	0	0	0	1	1	1	1	1	1	1	9
Franzblau et al. 2008, USA	0	0	1	0	0	0	1	1	1	1	1	1	1	8

## Discussion

This systematic review and meta-analysis examined the relationship between MBTs and mental health outcomes (PTSD symptoms, depressive symptoms, and anxiety symptoms) in women victims of violence. The results showed that MBTs were significantly associated with lower depressive symptoms, anxiety symptoms, and PTSD symptoms compared with active control or wait-list. The findings are consistent with systematic reviews and meta-analyses that MBTs are significantly effective in improving symptoms of depression, anxiety, and posttraumatic stress (D'Silva et al. 2012; Tan et al. 2023; Zhu et al. 2022). MBTs provide neuroplasticity, known as the ability of the brain to undergo structural or physiological changes. With neuroplasticity, MBTs may have affected neural networks that regulate emotional control, attention, mood, and executive functions and improved adverse mental health outcomes (Acevedo et al. 2016). Increased activation in the hypothalamus–pituitary–adrenal axis (HPA axis) and sympathetic nervous system (SNS) is observed with stress exposure. MBTs may have stabilized activation and improved negative mental health outcomes by acting as a regulator of increased activation of both HPA and SNS (Laird et al. 2019). The study result shows that limbic activation, which elicits stress responses, is deactivated during "OM" chanting used in meditation (Kanyani et al. 2011), the study result that yoga reduces sensitivity to cortisol hormone secreted as a result of increased HPA activation (Gothe et al. 2016); reveals the regulatory role of MBTs in HPA and SNS. Overall, the findings underline the importance of the use of MBTs in the treatment of anxiety, depression, and PTSD symptoms of women victims of violence.

The subgroup analysis provided a perspective on whether eight or fewer MBT sessions and more than eight MBT sessions compared to an active control or a wait-list made a difference in depression, anxiety, and PTSD symptoms in women victims of violence. Studies reporting PTSD, depression, and anxiety as mental health outcomes have focused on an average 8-week program (Mostafazadeh et al. 2019; Sharma and Rush 2014). To prevent the distribution of the number of sessions from being skewed heavily on one side and because the average number of sessions was eight, the limit of the distribution of the number of sessions was set at eight. Eight or fewer MBT sessions are more effective for anxiety and depression symptoms compared to active control or wait-list. However, no significant effect was found for PTSD symptoms. Our finding contradicts the results of the study that revealed a significant relationship between the total number of sessions of qigong, an MBT, and the reduction in anxiety and depression scores (Chan et al. 2014). The meta-analysis result that the total number of sessions of Baduanjin, an MBT, does not have a significant effect on anxiety level and that the depression level decreases with the increase in the total number of sessions does not support our finding (Zou et al. 2018). The fact that the group mindfulness-based stress reduction program and mindful yoga provided significant improvement in depression and anxiety at the end of 8 sessions supports our finding (Taleghani 2018). In a study conducted by Zhu et al. with patients with PTSD, mindfulness practice lasting 60–150 min per session for 8–16 weeks was found to be effective against other subgroups in improving PTSD symptoms (Zhu et al. 2022). The results should be interpreted with caution, as the number of sessions required to improve mental health symptoms may vary depending on the intervention, the severity of PTSD, and the characteristics of the patient population.

Mind–body therapies are significantly associated with lower anxiety, depression, and PTSD symptoms in women victims of violence. MBTs may contribute to positive mental health outcomes by improving the physiological changes caused by trauma-related stress. Therefore, interventions based on mind–body interaction can be used as an effective strategy for the rehabilitation of women victims of violence.

## Limitations

This study has some limitations. Although the interventions in the studies included in the meta-analysis were evaluated within the scope of mind–body therapies, the diversity of intervention types and durations may have changed the effect on mental health outcomes and prevented the identification of the effective MBT type. Subgroup analysis could not be performed because there were not enough randomized controlled trials to classify according to MBT type. This resulted in the inability to determine the effective MBT type. In addition, the included articles varied in terms of quality. Only 3 studies reported that the outcome assessors were blinded. In some studies, the randomization method was not described. These randomization and blinding issues may have affected the results of the study by reducing the reliability of the results and causing bias. Some studies did not adequately account for participants who dropped out of the study or examine whether dropouts made a difference in the analyses. This may reduce the reliability of the intervention or the validity of the study. In addition, the fact that the interventions varied in terms of time, frequency, duration and the outcome measurement tools used to evaluate the effectiveness of the intervention were different in the studies may lead to different results in mental health criteria and may cause difficulties in explanation. In some studies, no intervention was applied to the control group. This makes it difficult to assess whether mind–body therapies have a specific effect.

Although this study reported significant associations between MBT and mental health outcomes (PTSD symptoms, depressive symptoms, and anxiety symptoms) in women victims of violence, there was high heterogeneity between the studies. The exposure of the study population to different types of violence in different settings, the variety of measurement instruments, and differences in the frequency and timing of follow-up testing may have contributed to the high heterogeneity. The included studies used different self-report scales to measure anxiety and depression symptoms. Self-report scales may have contributed to heterogeneity as they are subjective measures based on individuals' perceptions and interpretations of their symptoms.

The included studies have methodological and conceptual limitations. The included studies were spread across North America, Oceania, Europe and Asia. However, the included studies provided diversity in terms of sociodemographic factors such as race or ethnicity, income, education, employment status and marital status. Although we can generalize the results of the study across continents, the results are limited in terms of generalisability due to racial, socioeconomic, and marital status. The use of MBTs varies among different racial and ethnic groups (Hsiao et al. 2006). Cultural background may limit the use of MBTs by significantly affecting attitudes and beliefs towards these therapies.

## Clinical implications

CBT is a widely recognised and effective treatment for PTSD (Mueser et al. 2008; Surís et al. 2013). On the other hand, mind–body practices have also been found to be a suitable intervention to improve PTSD symptoms, including intrusive memories, avoidance, and emotional arousal (Kim et al. 2013). A study on the effects of mind–body exercises on PTSD symptoms, depression and anxiety in PTSD patients also emphasised the potential benefits of mind–body interventions in PTSD treatment (Zhu et al. 2022). The effectiveness of mindfulness-based cognitive therapies in reducing avoidance symptoms and PTSD cognitions suggests that MBTs show promise as brief interventions (King et al. 2013). Considering that a larger number of sessions in the treatment of PTSD may cause drop-outs (Imel et al. 2013), MBTs can be used as short-term interventions in women victims of violence.

## Conclusion

This study shows that mind–body therapies have a positive effect on anxiety, depression, and PTSD symptoms in women victims of violence when compared with active control or wait-list. The clinical studies comparing MBTs with leading therapy modalities in the treatment of PTSD are needed. Thus, limitations and strengths regarding the application of MBTs should be revealed.

## Data Availability

Data from this meta-analysis is available upon request.
